# Advanced MRI features of intraventricular inflammatory myofibroblastic tumor: a case report

**DOI:** 10.1186/s12883-022-02993-8

**Published:** 2022-12-02

**Authors:** Xiao-lin Tang, Wen-jun Huang, Qiang Ma, Kun-ming Yi

**Affiliations:** 1 Department of Radiology, Daping Hospital, Army Medical University, Chongqing, 400042 China; 2 Chongqing Clinical Research Centre of Imaging and Nuclear Medicine, Chongqing, 400042 China; 3 Department of Radiology, Traditional Chinese Medicine Hospital of Banan District, Chongqing, 401320 China; 4 Department of Pathology, Daping Hospital, Army Medical University, Chongqing, 400042 China

**Keywords:** Inflammatory myofibroblastic tumor, Intraventricular, Magnetic resonance imaging

## Abstract

**Background:**

Inflammatory myofibroblastic tumor (IMT) is a rare central nervous system (CNS) tumor. We first report a rare case of IMT in the lateral ventricle and describe the magnetic resonance imaging (MRI) findings of the tumor with an emphasis on the advanced MRI features.

**Case presentation:**

A 49-year-old female patient with headaches and blurred vision for 2 months. Brain MRI revealed a well-circumscribed, lobulated mass occupying the left lateral ventricle trigone, with marked perilesional brain edema. The tumor showed heterogeneous significant hyperintensity on T2-weighted imaging (T2WI) and hypointensity on T1-weighted imaging (T1WI). After the administration of gadolinium, the mass exhibited marked contrast enhancement and the halo sign was observed. On advanced MRI, the lesion showed decreased perfusion on perfusion MRI and reduced diffusion on diffusion-weighted imaging (DWI). On susceptibility-weighted imaging (SWI), there was a punctate low signal intensity in the tumor. The patient underwent surgical resection of the mass and a pathological examination confirmed the lesion to be an inflammatory myofibroblastic tumor with negative expression of anaplastic lymphoma kinase (ALK). This patient had remained healthy without evidence of recurrence during a 20-month follow-up.

**Conclusions:**

On MRI, marked perilesional brain edema, significant hyperintensity on T2WI, hypoperfusion on perfusion MRI but with an obvious enhancement, no diffusion restriction on DWI, and halo sign may be the characteristic findings of intraventricular IMT. The advanced MRI characteristics could provide abundant information to reflect the histological features and physiological metabolic characteristics of the tumor.

**Supplementary Information:**

The online version contains supplementary material available at 10.1186/s12883-022-02993-8.

## Background

Inflammatory myofibroblastic tumor (IMT), which was officially named by the World Health Organization in 2002, is an intermediate fibroblastic /myofibroblastic tumor with incompletely known etiology and pathogenesis [1]. Histologically, IMT is composed of myofibroblastic mesenchymal spindle cells accompanied by an inflammatory infiltrate of plasma cells, lymphocytes, and eosinophils [2]. IMT commonly occurs in children and adolescents. IMT could arise in any part of the body, the lung is the most common site, followed by mesenteric, omental, and retroperitoneal, but rarely develop in the intracranial [3]. In the central nervous system (CNS), IMT can occur in the meninges, cerebral hemisphere, cerebellar hemisphere, skull base, and scalp [3–9]. Häusler et al. [4] classified intracranial IMT into 5 types according to anatomic distribution, including meningeal lesions, intraparenchymatous lesions, mixed intraparenchymatous lesions and meningeal lesions, intraventricular lesions, and lesions extending between intra- and extracerebral sites. Nearly 60% of the intracranial IMTs originated from the meninges and 12% of them originated from the brain parenchyma [4]. The clinical symptoms of intracranial IMT are nonspecific.

In this study, we first report a case of IMT in the lateral ventricle and describe the advanced MRI characteristics of the tumor, including characteristics observed on diffusion-weighted imaging (DWI), apparent diffusion coefficient (ADC), diffusion tensor imaging (DTI), susceptibility-weighted imaging (SWI), MR spectroscopy (MRS), and dynamic susceptibility-weighted contrast-enhanced perfusion (DSC perfusion). These advanced image findings have not been reported before.

## Case presentation

**Fig. 1:**
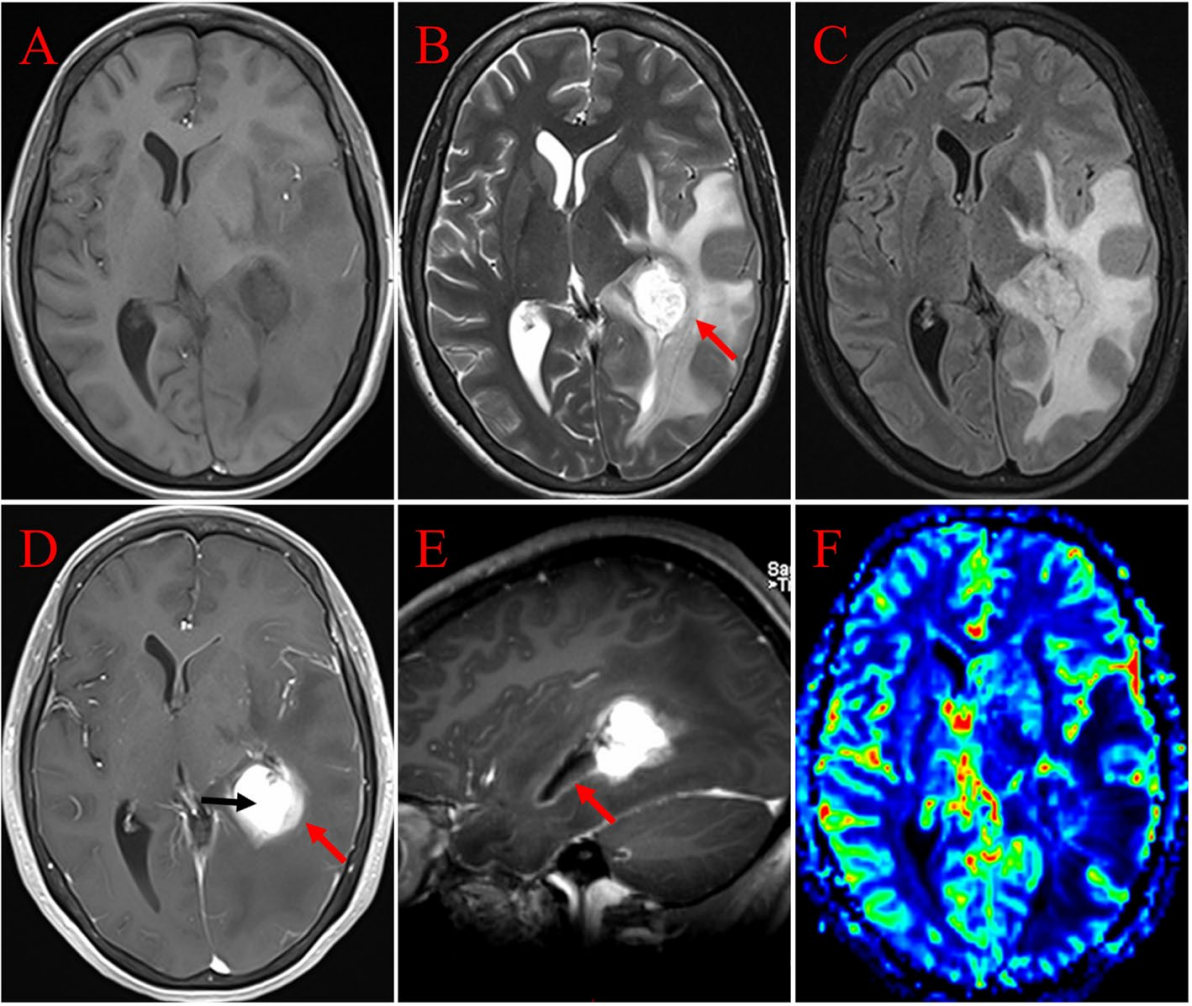
MRI shows a well-circumscribed, lobulated mass in the left lateral ventricle trigone, with marked perilesional brain edema. **A** On the axial T1WI, the lesion displays low to intermediate signal intensity compared to that of the gray matter. **B** On the axial T2WI, the tumor displays heterogeneous high signal intensity with a few hypointense areas, and a low signal ring surrounding the neoplasm (arrows). **C** On the axial FLAIR, the lesion shows heterogeneous hyperintensity. **D** On the axial contrast-enhanced MRI, the tumor demonstrates significant enhancement and appears with the halo sign, the distinctive halo (red arrows) shows relatively weak enhancement compare to that of the mass (black arrows). **E** On the sagittal contrast-enhanced MRI, the lateral ventricular wall showed enhancement (red arrows). **F** On the corresponding color CBV image, the tumor shows decreased perfusion

**Fig. 2:**
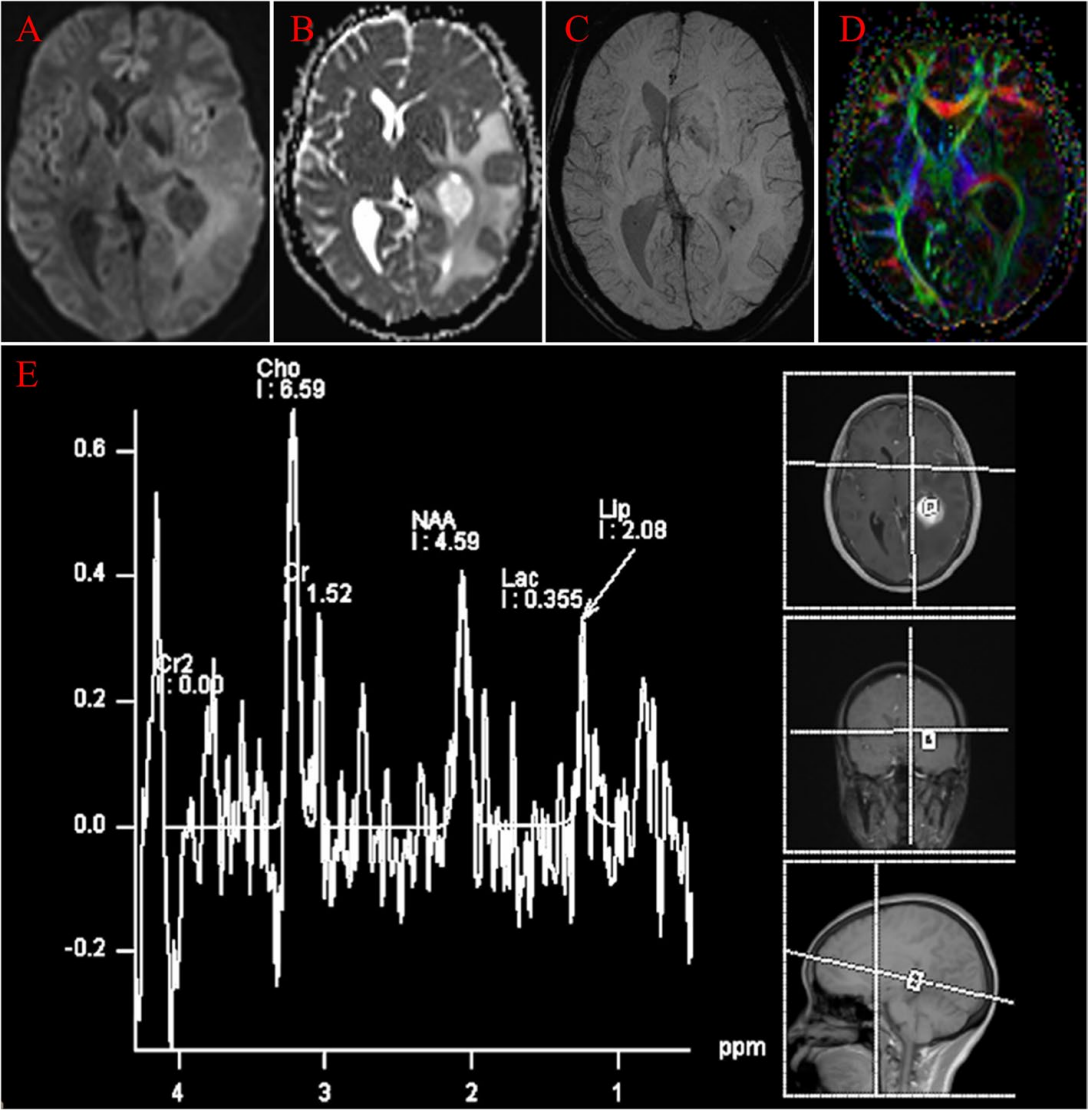
**A**, **B** On the axial DWI and correlative ADC map, the tumor displays hypointensity and hyperintensity, respectively. **C** On SWI, the lesion shows a little punctate low signal intensity. **D** On the fractional anisotropy (FA) color map, the adjacent white matter fiber tracts were not damaged by the pressure of the tumor. (**E**) On multi-voxel proton MRS, the lesion shows decreased NAA, increased Cho value, and increased Lac/Lip value

A 49-year-old woman with no significant past medical or family history was admitted to our hospital due to headaches and blurred vision for 2 months. Neurological and laboratory investigations showed no positive findings. Brain MRI scans (Magnetom Verio 3.0 T; Siemens, Germany) demonstrated a 2.8 cm × 2.9 cm × 2.8 cm well-circumscribed, lobulated mass in the left lateral ventricle trigone, with marked perilesional brain edema. On T1-weighted imaging (T1WI), the lesion presented with heterogeneous slight hypointensity compared to that of the gray matter (Fig. 1A). On both T2-weighted imaging (T2WI) and fluid-attenuated inversion recovery (FLAIR), the mass showed marked hyperintensity with a few areas of low signal intensity (Fig. 1B, C). After the administration of gadolinium, the mass demonstrated intense enhancement and the adjacent lateral ventricular wall also showed enhancement (Fig. 1D, E). In addition, the gadolinium-enhanced MRI showed the lesion with a halo sign—a distinctive zone or halo surrounding the mass, having a relatively weak enhancement than that of the mass (Fig. 1D). On perfusion MRI, the tumor showed decreased perfusion, both relative cerebral blood volume (rCBV) (Fig. 1F) and relative cerebral blood flow (rCBF) were relatively reduced. The tumor without diffusion restriction on DWI (Fig. 2A) and with an elevated ADC value (rADC_min_ = 2.9) on the correlative ADC map (Fig. 2B). On SWI, a little punctate low signal intensity was found in the tumor (Fig. 2C), suggesting the absence of bleeding or calcification within the tumor. A fractional anisotropy (FA) color map showed the adjacent white matter fiber tracts were not damaged by the pressure of the tumor (Fig. 2D). On multi-voxel proton MRS, the tumor region showed elevated choline (Cho) and lower levels of N-acetylaspartate (NAA), increased Lac/Lip value also can be found (Fig. 2E). On MRI, no cysts or necrosis were identified in the tumor.

**Fig. 3:**
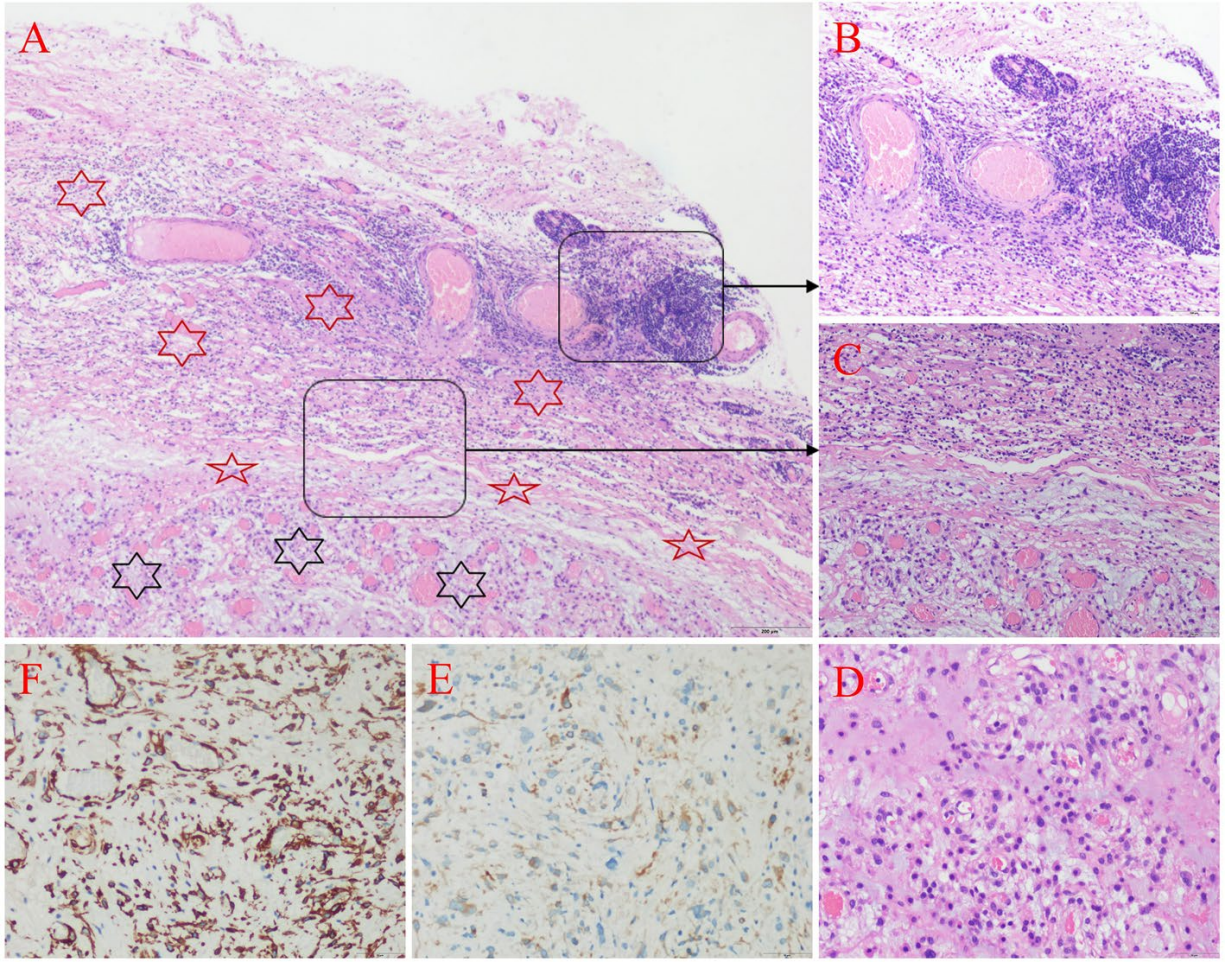
Histology and immunohistochemistry of inflammatory myofibroblastic tumor. **A** Hematoxylin and eosin staining demonstrates that there is a clear demarcation between tumor tissue (black hexagonal star) and peritumoral tissue (red hexagonal star), and the tumor has a relatively complete pseudocapsule (red five-pointed star) (hematoxylin and eosin, × 40). **B** In the peritumoral tissue, there are abundant blood vessels and infiltration of inflammatory cells. Some regions of lymphocytes aggregate to form follicle-like structures, and some areas of lymphocytes surround the blood vessels to form cuff-like changes (hematoxylin and eosin, × 100). **C** The pseudocapsule is composed of proliferative fibrous tissue with lymphocyte infiltration (hematoxylin and eosin, × 100). **D** In the mucinous background, the tumor cells are distributed in bundles and surround blood vessels. The tumor cells are round or oval, with eosinophilic cytoplasm and small nucleoli (hematoxylin and eosin, × 200). **E** Immunostaining for ALK-1 is weakly positive (× 200). **F** Immunostaining for Caldesmon is diffuse positive (× 200). Type of equipment for all microscopy images: microscope: Olympus BX43; objective: Olympus Plan N; camera: Olympus DP26, DP26-CU, SN1120809; software: Olympus cellSens Entry

The patient underwent surgical resection of the mass. Histopathologically, spindle myofibroblasts proliferated and distributed in fascicles in a mucous background. Tumor cells were round or oval, with eosinophilic cytoplasm and small nucleoli. Mitotic figures could be seen in some areas. There were abundant blood vessels and a large number of inflammatory cells in the tumor stroma. The tumor had a clear boundary with the surrounding tissue and had a relatively complete pseudocapsule in which inflammatory cells were infiltrated. In the peritumoral tissue, blood vessels were hyperplasia and dilated, and inflammatory cells were infiltrated. Immunohistochemical staining revealed the following: Anaplastic lymphoma kinase1 (focal positive), Calponin (+), Caldesmon (+), Desmin (focal positive), Nestin (+), Vimentin (+), Cluster of differentiation 34 (+), Integrase interactor 1(retained), Smooth muscle actin (−), Cluster of differentiation 10 (−), Cytokeratin (−), Epithelial membrane antigen (−), Glial fibrillary acidic protein (−), Isocitrate dehydrogenase 1 (−), Myoblast *determination protein* 1(−), S-100 protein (−), Myogenin (−), Olig-2 (−), Sata6 (−). The tumor had a Ki-67 proliferation index of 3%. Although molecular testing showed that the anaplastic lymphoma kinase (ALK) gene of the tumor did not undergo breakage and recombination, the histological and immunohistochemical findings were consistent with a diagnosis of IMT (Fig. 3, Additional file 1). The postoperative MRI scan confirmed that the tumor had been completely removed, and after 20 months of follow-up, there was no recurrence.

## Discussion and conclusion

Inflammatory myofibroblastic tumor is characterized by myofibroblastic spindle cell proliferation with inflammatory cell infiltration. Immunohistochemistry analysis could show positive staining for anaplastic lymphoma kinase, smooth muscle actin, muscle-specific actin, desmin, calponin, vimentin, and cytokeratin [2]. Microscopically, IMTs can be divided into 3 growth patterns: the nodular fasciitis-like variant, the fibromatosis/fibrohistiocytic type, and the desmoid type [1]. Hence a diagnosis of IMT was established in our case according to the histological and immunohistochemical findings. Meanwhile, we think the presented case falls into the category of the nodular fasciitis-like variant because this case had abundant mucus, blood vessels, and inflammatory cells.

In this study, we summarized the characteristics of conventional MRI and functional imaging of a patient with intraventricular IMT. On conventional MRI imaging, the presented case manifested as a well-circumscribed, lobulated lesion, with hypointensity on T1WI and hyperintensity on both T2WI and T2WI-FLAIR. The lesion showed marked enhancement after gadolinium administration. These conventional imaging findings correlate well with the pathological changes that the tumor is rich in blood vessels and mucus. However, the conventional MRI characteristics of IMT are nonspecific and cannot be distinguished from other tumors of the central nervous system, such as meningioma, lymphoma, and high-grade glioma. In our study, the advanced MRI manifestations of the intraventricular IMT were analyzed in detail, including DWI, ADC values, FA color map, MRS, DSC perfusion, and SWI manifestations, these advanced imagings provide more information related to the histological features and physiological metabolic characteristics of the tumor, such as vascularity, cellularity, and mitotic indices.

DWI with ADC measurements is a reliable diagnostic technique that provides information regarding the diffusion of water molecules and partly reflects tissue cellularity. The increase in tumor cellularity increases nucleus-to-cytoplasm ratios and decreases extracellular space, leading to restricted water molecules diffusion. In CNS tumors, due to their high cellularity, high-grade malignant tumors usually present lower ADC compared to low-grade tumors. In general, hypercellular tumors tend to show hyperintensity on DWI with a low ADC [10]. In the present case, On DWI, the mass showed low signal intensity with a high ADC value (rADC_min_ = 2.9). These image characteristics correspond to the histopathological features of IMT, such as abundant myxoid stroma and relatively few cellular components. For another, these MRI findings collectively suggested that the tumor shows the histological characteristics of a benign or low-grade malignant tumor rather than a high-grade malignant tumor.

SWI is an effective noninvasive technique for detecting bleeding and calcification and shows low signal intensity in the presence of these artifacts [11]. On SWI, the tumor did not show a lot of low signals, suggesting the absence of significant bleeding and calcification, which was consistent with the histological findings.

Proton MRS is a valuable technique for reflecting the metabolism of tumors. Choline (Cho) can reflect the production and repair of myelin of the cell membrane, so the elevated levels of Cho represent cell proliferation or increased metabolism. N-acetyl-aspartate (NAA) is generally recognized as a sign of functioning neurons, the decrease of NAA in intracranial tumors may indicate a decrease or destruction of nerve cells [12]. The current case demonstrated decreased NAA, increased Cho value and Cho/NAA ratio, the average Cho/NAA value was 2.19. Increased lactate (Lac) and lipid (Lip) also can be found in this case. However, these results of the tumor were not specific.

Dynamic susceptibility-weighted contrast-enhanced perfusion (DSC-PWI) is an important tool to evaluate the vascular function and structure changes of the tumor by using tumor hemodynamic information and indirectly reflecting the degree of tumor malignancy [13]. In this case, the lesion demonstrated marked enhancement, but both relative cerebral blood volume (rCBV) and (relative cerebral blood volume) rCBF were reduced on DSC-PWI, indicating decreased perfusion, these features are similar to the performance of lymphoma. Histologically, the tumor showed capillary hyperplasia and vasodilation, and some of the blood vessels were filled with red blood cells. Therefore, we suggested that IMT appeared avid enhancement may be due to the breakdown of the blood-brain barrier, abnormally permeable capillaries, and vascular hyperplasia. Although our histopathological findings confirmed that the lesion has much neovascularization, maybe the relatively low microvascular area results in low perfusion.

In this case, on gadolinium-enhanced MRI, the tumor appeared with the CT halo sign which represents a zone of lower enhancement surrounding the mass. Interestingly, the signal intensity of the distinctive zone was lower than that of the mass on T2WI and FLAIR. Histologically, we demonstrated the tumor showed well-circumscribed with pseudocapsule, and the adjacent brain tissue without obvious invasion but with capillary dilation and perivascular inflammatory cells infiltrating. Consequently, we considered that the marginal areas are hyperemic cerebral tissues with inflammatory cells infiltrating not part of the tumor, and abnormal vascular permeability is the main reason for marginal zone enhancement.

In conclusion, intraventricular IMT is an uncommon central nervous system tumor. On MRI, marked perilesional brain edema, significant hyperintensity on T2WI, no diffusion restriction on DWI, hypoperfusion but an obvious enhancement, and halo sign may be the characteristic manifestations of intraventricular IMT. The advanced MRI characteristics could provide abundant information related to the histological features and physiological metabolic characteristics of the tumor. Certainly, further large-scale studies are warranted to confirm these characteristics. Despite the final diagnosis depending on the histopathologic and immunohistochemical analysis, we believe that combining the conventional MRI with advanced MRI is useful for the preoperative diagnosis and differentiation of intraventricular IMT from other tumors and tumor-like lesions.

## Supplementary Information

**Additional file 1: **Histopathology of inflammatory myofibroblastic tumor. **Fig. S1, Fig. S2.** Photomicrographs show that the tumor cells are round or oval, with eosinophilic cytoplasm and small nucleoli. The tumor cells are diffusely distributed with scattered lymphocyte infiltration. There are abundant small blood vessels and myxoid changes in the tumor stroma (hematoxylin and eosin, × 100). **Fig. S3.** At the junction of the tumor and normal tissue, a large number of lymphocytes infiltrate, and many muscle fibers are arranged in bundles. Dilated small blood vessels are present in the stroma (hematoxylin and eosin, × 100). **Fig. S4.** Photomicrograph shows that many blood vessels and scattered lymphocytes in the mucinous background, and some tumor cells surround blood vessels (hematoxylin and eosin, × 200). Type of equipment for all microscopy images: microscope: Olympus BX43; objective: Olympus Plan N; camera: Olympus DP26, DP26-CU, SN1120809; software: Olympus cellSens Entry. All pathological images were acquired with a uniform measured resolution of 2448 × 1920 pixels.

## Data Availability

The datasets generated during the current study will be available in the Biological Magnetic Resonance Data Bank (https://bmrbig.org/released/bmrbig71).

## Abbreviations

IMT: Inflammatory myofibroblastic tumor
CNS: Central nervous system
MRI: Magnetic resonance imaging
T1WI: T1-weighted imaging
T2WI: T2-weighted imaging
DWI: Diffusion-weighted imaging
SWI: Susceptibility-weighted imaging
MRS: Magnetic resonance spectroscopy
DSC-PWI: Dynamic susceptibility contrast-enhanced perfusion-weighted imaging
ADC: Apparent diffusion coefficient
Cho: Choline
NAA: N-acetylaspartic acid
Lac: Lactate
Lip: Lipid
rCBV: Relative cerebral blood volume
rCBF: Relative cerebral blood flow
FLAIR: Fluid-attenuated inversion recovery
