# Supplementary figures and images for: Advanced MRI features of intraventricular inflammatory myofibroblastic tumor: a case report
Source: BMC Neurol. 2022 Dec 2;22:448. doi: 10.1186/s12883-022-02993-8 (PMC9717528; doi:10.1186/s12883-022-02993-8)

Figure S1

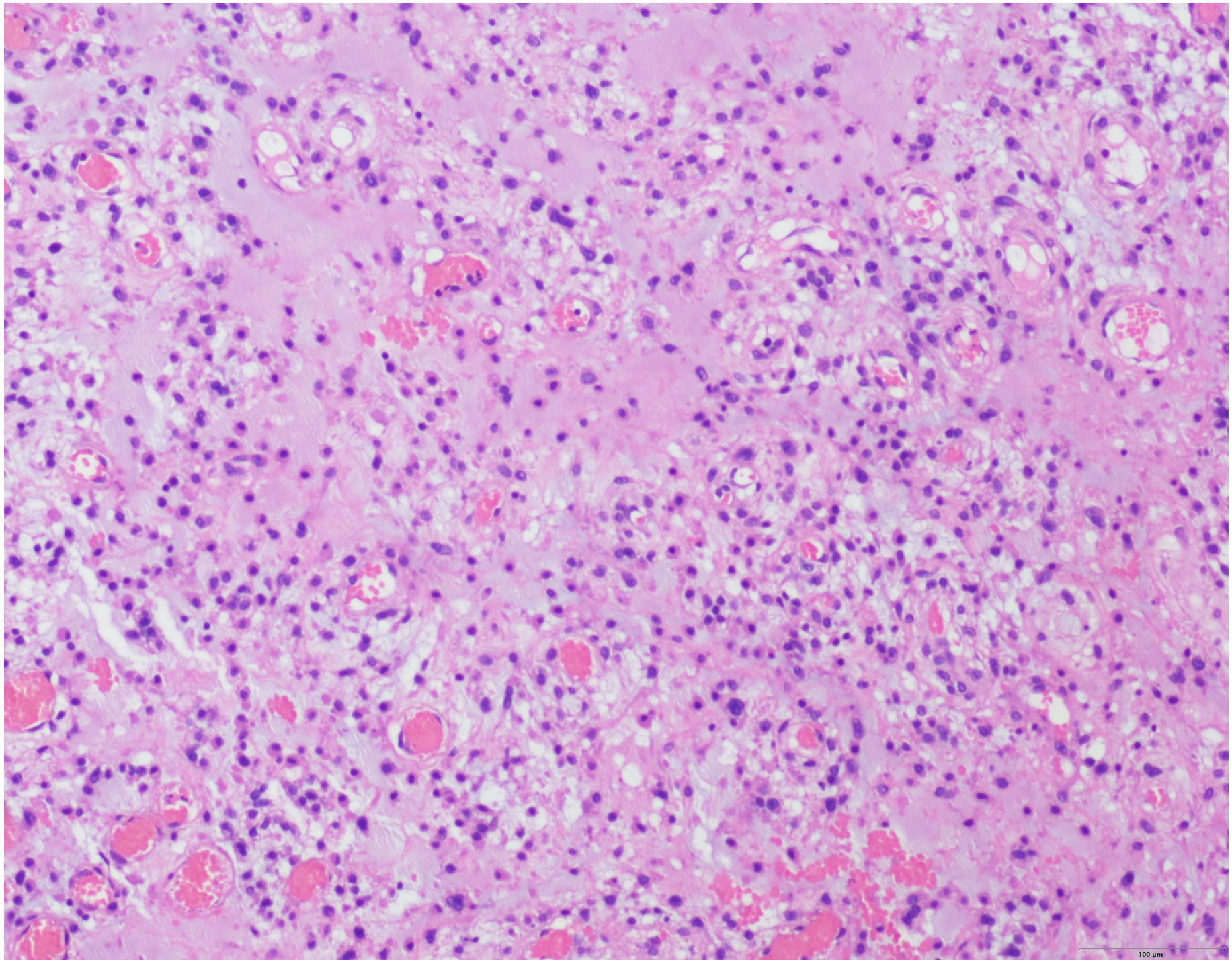

Figure S2

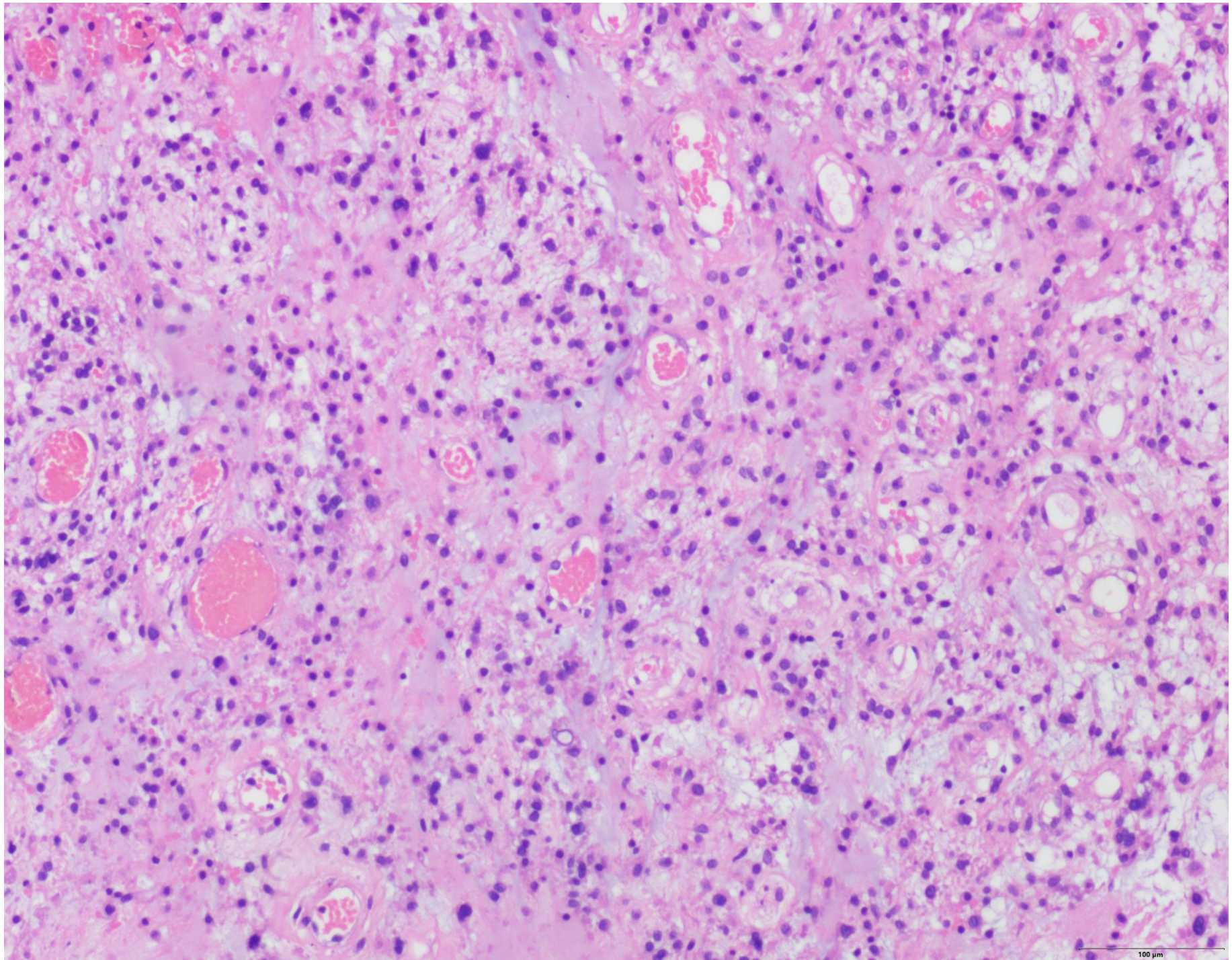

Figure S3

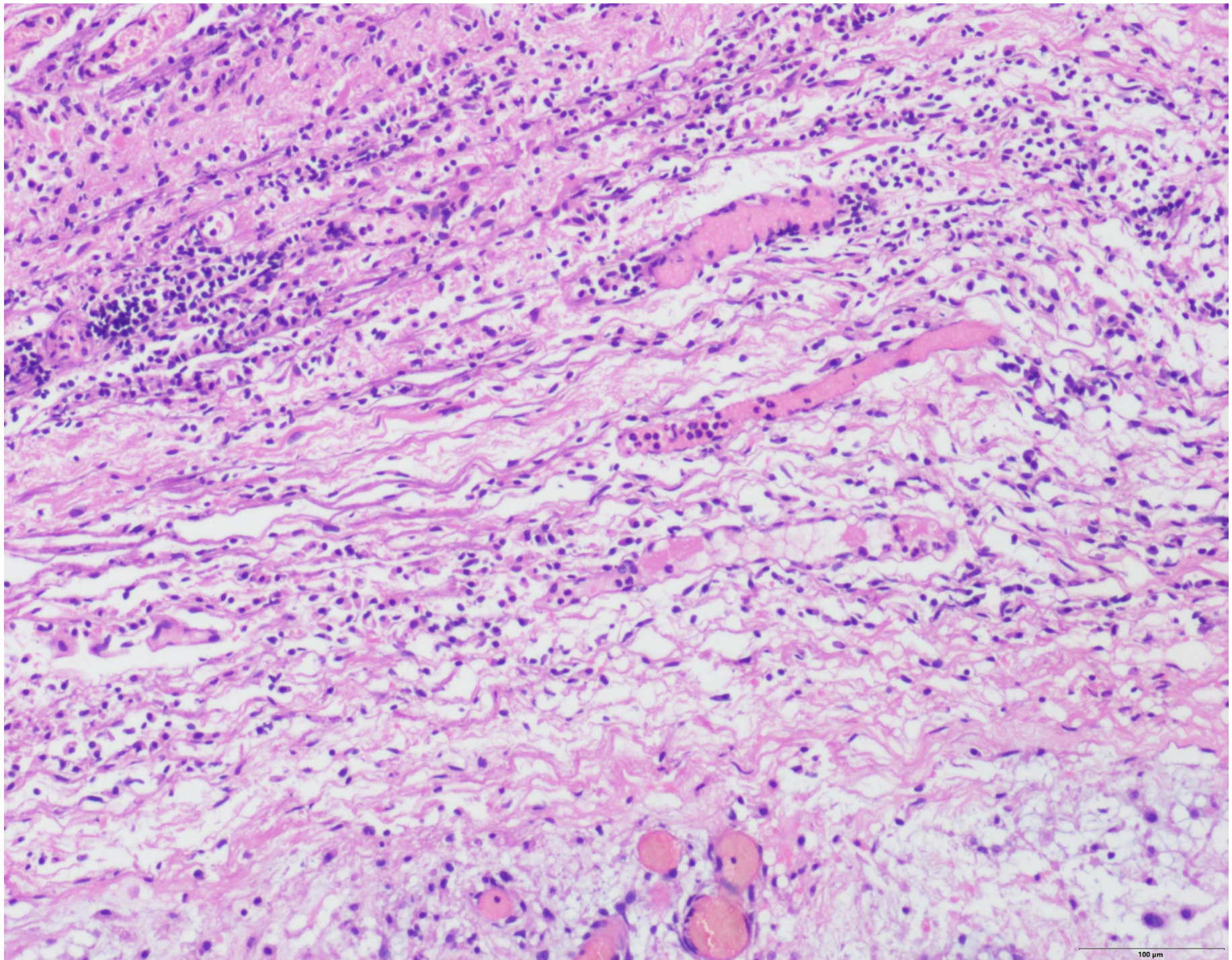

Figure S4

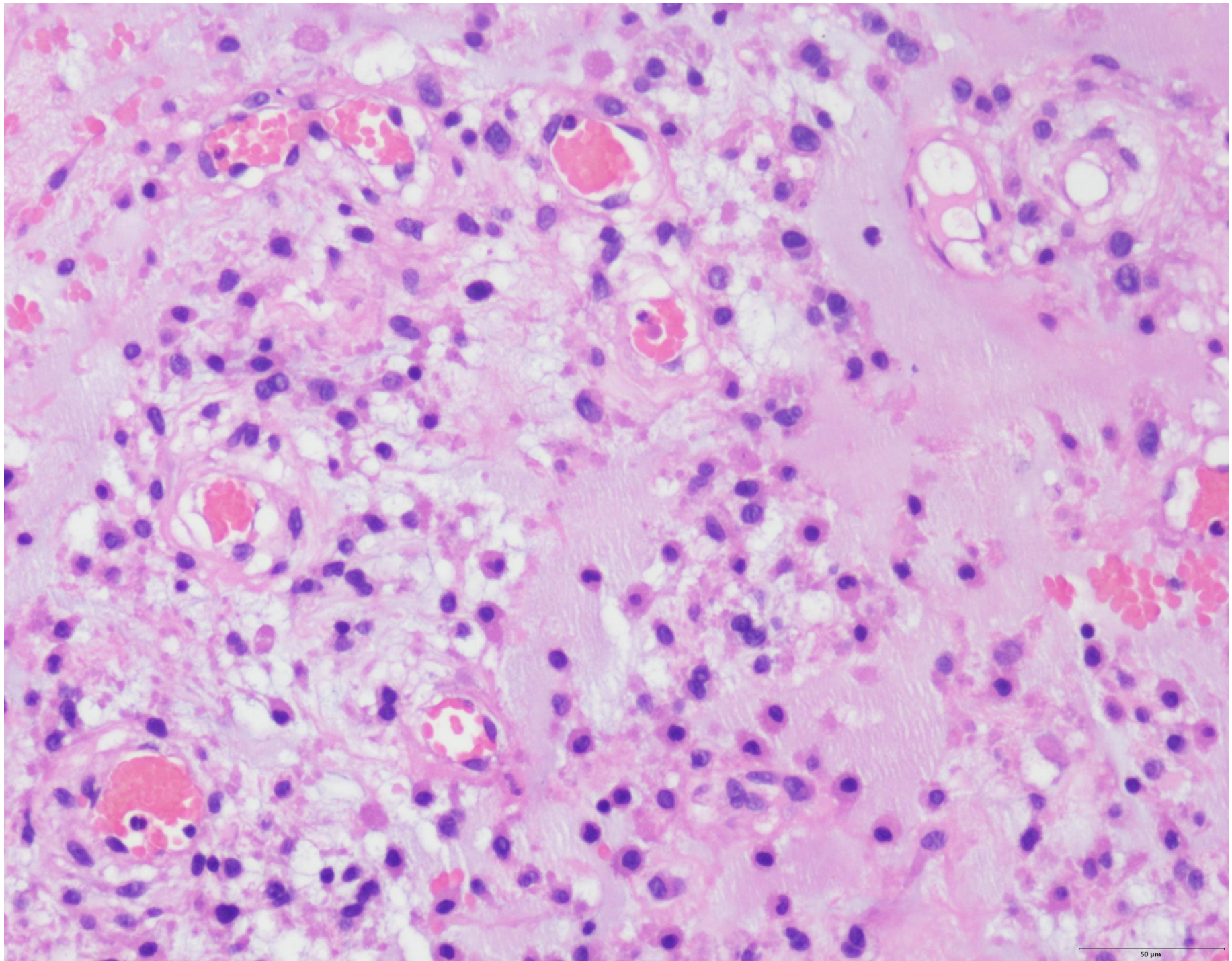

Supplement: Supplementary file 1 — Additional file 1: Histopathology of inflammatory myofibroblastic tumor. Fig. S1, Fig. S2. Photomicrographs show that the tumor cells are round or oval, with eosinophilic cytoplasm and small nucleoli. The tumor cells are diffusely distributed with scattered lymphocyte infiltration. There are abundant small blood vessels and myxoid changes in the tumor stroma (hematoxylin and eosin, × 100). Fig. S3. At the junction of the tumor and normal tissue, a large number of lymphocytes infiltrate, and many muscle fibers are arranged in bundles. Dilated small blood vessels are present in the stroma (hematoxylin and eosin, × 100). Fig. S4. Photomicrograph shows that many blood vessels and scattered lymphocytes in the mucinous background, and some tumor cells surround blood vessels (hematoxylin and eosin, × 200). Type of equipment for all microscopy images: microscope: Olympus BX43; objective: Olympus Plan N; camera: Olympus DP26, DP26-CU, SN1120809; software: Olympus cellSens Entry. All pathological images were acquired with a uniform measured resolution of 2448 × 1920 pixels. [file 12883_2022_2993_MOESM1_ESM.pdf]
